# A Shift in Policy? Learning from China’s Environmental Challenges and Successes

**DOI:** 10.1289/ehp.119-a307b

**Published:** 2011-07-01

**Authors:** M. Nathaniel Mead

**Affiliations:** M. Nathaniel Mead, a science writer living in Durham, NC, has written for *EHP* since 2002.

Outdoor air pollution in Hong Kong SAR, Beijing, and China’s other major urban centers far exceeds international health-based standards, and the top Chinese environmental regulator has classified more than half the country’s water resources as too polluted for human use. Yet in 2009 China became the world’s number one investor in clean energy technology, investing nearly twice as much as the United States. The situation is emblematic of China’s paradoxical nature: Although this country has routinely pursued policies that promote economic growth at the expense of environmental health, in more recent years it has begun to make commitments to environmental protection, reaching milestones that set examples for U.S. and other Western policy makers  [*EHP* 119(7):893–895; Remais and Zhang].

China has multiple incentives to invest in energy efficiency and alternative energy sources, including the large numbers of premature deaths linked to outdoor and indoor air pollution from highly polluting energy sources. The country has adopted strict national fuel efficiency standards for vehicles that widely exceed U.S. requirements and has paired these standards with tailpipe emissions controls that reduce traffic-related air pollution. In a bid to reduce its reliance on coal and other fossil fuels, China has increased its renewable energy production capacity by 79% since 2005 (compared with a 24% increase in the United States during the same period) and set ambitious targets to produce more than 15% of its electricity supply from renewable sources by 2020.

**Figure:**
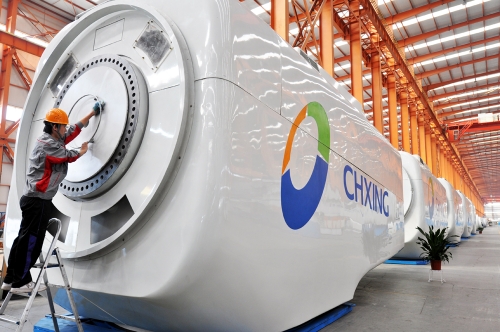
A Chinese worker checks a wind turbine at a plant in Zouping County, Shandong Province. © Imaginechina via AP Images

The authors trace China’s recent progress to a surge in investments since 1991 aimed at improving environmental quality. These investments have led to increased enforcement of, and improved compliance with, existing laws governing pollution abatement, resource conservation, and ecological management. Last year, the Chinese National People’s Congress issued a $20-billion plan for new energy technologies, ambitious energy conservation measures, and environmental protection initiatives.

But to make a comprehensive environmental protection framework really work in China, the authors contend the country must reassess its position on economic growth as the prime driver behind policy making. China must also address the dual role of the government as both regulator and polluter as well as strive for greater transparency and enforcement in environmental matters. “Progress in these regulatory areas will grant the country greater influence on the world stage,” the authors write. Even now, however, the authors assert China has lessons to offer Western policy makers as global leaders work to limit the serious environmental externalities that accompany reliance on fossil fuels.

